# Bipolar disorders and schizophrenia: discrete disorders?

**DOI:** 10.3389/fpsyt.2024.1352250

**Published:** 2024-04-30

**Authors:** Micaela Dines, Mariana Kes, Delfina Ailán, Marcelo Cetkovich-Bakmas, Christoph Born, Heinz Grunze

**Affiliations:** ^1^Department of Psychiatry, Instituto de Neurología Cognitiva (INECO), Buenos Aires, Argentina; ^2^Department of Psychiatry, Instituto de Neurociencia Cognitiva y Traslacional (Consejo Nacional de Investigaciones Científicas y Técnicas - Fundación INECO - Universidad Favaloro), Buenos Aires, Argentina; ^3^Department of Psychiatry, Psychiatrie Schwäbisch Hall, Ringstraße, Germany; ^4^Department of Psychiatry, Paracelsus Medical University, Nuremberg, Germany

**Keywords:** bipolar disorder, genetics, psychosis, schizophrenia, spectrum disorder

## Abstract

**Background:**

With similarities in heritability, neurobiology and symptomatology, the question has been raised whether schizophrenia and bipolar disorder are truly distinctive disorders or belong to a continuum. This narrative review summarizes common and distinctive findings from genetics, neuroimaging, cognition and clinical course that may help to solve this ethiopathogenetic puzzle.

**Methods:**

The authors conducted a literature search for papers listed in PubMed and Google Scholar, using the search terms “schizophrenia” and “bipolar disorder” combined with different terms such as “genes”, “neuroimaging studies”, “phenomenology differences”, “cognition”, “epidemiology”. Articles were considered for inclusion if they were written in English or Spanish, published as full articles, if they compared subjects with schizophrenia and bipolar disorder, or subjects with either disorder with healthy controls, addressing differences between groups.

**Results:**

Several findings support the hypothesis that schizophrenia and bipolar disorder are discrete disorders, yet some overlapping of findings exists. The evidence for heritability of both SZ and BD is obvious, as well as the environmental impact on individual manifestations of both disorders. Neuroimaging studies support subtle differences between disorders, it appears to be rather a pattern of irregularities than an unequivocally unique finding distinguishing schizophrenia from bipolar disorder. The cognitive profile displays differences between disorders in certain domains, such as premorbid intellectual functioning and executive functions. Finally, the timing and trajectory of cognitive impairment in both disorders also differs.

**Conclusion:**

The question whether SZ and BD belong to a continuum or are separate disorders remains a challenge for further research. Currently, our research tools may be not precise enough to carve out distinctive, unique and undisputable differences between SZ and BD, but current evidence favors separate disorders. Given that differences are subtle, a way to overcome diagnostic uncertainties in the future could be the application of artificial intelligence based on BigData.

**Limitations:**

Despite the detailed search, this article is not a full and complete review of all available studies on the topic. The search and selection of papers was also limited to articles in English and Spanish. Selection of papers and conclusions may be biased by the personal view and clinical experience of the authors.

## 1 Introduction

Bipolar disorder (BD), as proposed by Kleist and Leonhard, is nowadays used to refer to Kraepelin´s Manic Depressive Illness. BD and schizophrenia (SZ), in spite of having a clear phenomenology, share some phenomenological manifestations that often makes differential diagnosis difficult. Since Emil Kraepelin described fundamental aspects that distinguished “manic-depressive illness” from “dementia präcox”140 years ago, countless scientific studies have been carried out that have failed to establish the unique etiopathogenic element that defines each of them (1). Instead, a dimensional approach that conceives them as the extremes of a continuum has been put forward by several distinguished researchers (2–4), reviving the concept of a unitary psychosis as it has been put forward by 19^th^ century researchers prior to Kraepelin’s dichotomy According to this concept, there were no distinct disease entities in psychiatry but only varieties of a single universal madness, and the boundaries between these variants were fluid (5).Despite the large amount of research on the subject, however, there is no solid evidence to indicate that the Kraepelinian dichotomous categorical model should be abandoned (6). Instead, there is reasonable evidence to support their individual entity. A unique and discriminable psychopathology based on clinical symptom profile, outcome and prognosis as well as family history data constitutes a working hypothesis sustained for more than a century by schools such as Wernicke-Kleist-Leonhard (7). Not being the dominant approach in the clinic, Leonhard’s nosology has encountered difficulties in producing convincing data that generates wide acceptance (8). More recently, however, analysis of large databases comparing categorial diagnosis and polygenetic risk scores also favors SZ and BD being distinctive entities (9).

Both SZ and BD are severe disorders, affecting the same organ, the brain, and having several commonalities in symptomatology and outcome. Yet, not only for sophisticated reasons, but for the sake of patients who need optimized treatment, SZ and BD need to be conceptualized as independent disorders. To illustrate the matter: Declining function and dyspnea affect the same organ (lung) but may be the result of a multitude of different diseases, e.g. allergic asthma, COPD, pulmonary edema, to name a few. Just labelling it as “Unitary pulmonary disease” would not be very helpful. Without a firm diagnosis, causal treatment cannot be established, but only unspecific support (e.g., oxygen supply) can be offered.

In this narrative review, we will examine the current evidence that supports the hypothesis that there are distinctive boundaries separating SZ from BD. Carving out the often subtle differences will foster future research for individual and optimized treatment of BD and SZ, while resting on a dimensional concept of an unitary psychosis is likely to lay scientific curiosity and progress to rest.

## 2 Methods

The aim of this article is to review the main characteristics that distinguish SZ from BD. This narrative review by six authors with clinical experience in the field is based on selected papers, retrieved from PubMed and Google Scholar. Search term applied were: “(genetic differences) AND (schizophrenia) AND (bipolar disorder); (neurobiological differences) AND (schizophrenia) AND (bipolar disorder); (clinical differences) AND (schizophrenia) AND (bipolar disorder); (differentiating bipolar disorder type I) AND (schizophrenia); (differentiating schizophrenia) AND (bipolar disorder); (schizophrenia) AND (bipolar) AND (progression).

Our initial PubMed search on June 30, 2023 for systematic reviews or meta-analyses on commonalities and differences between SZ and BD returned 80 unique records. Repetition of the search in Google scholar did not add any additional hits. Of these reports, 54 met our predefined inclusion criteria. We excluded 26 studies as ineligible. Studies were considered for exclusion if they were not published as full articles, or if they focused on disorders other than SZ and BD. Moreover, we excluded records if they mainly focused on highly specific aspects of SZ/BD that were beyond the scope of our review, or have not been reproduced, or are of very limited clinical utility (e.g., retinal biomarkers; serum repetin levels). Subsequently, the selected 54 articles referred to 86 additional PubMed listed reports supplying information and research results cited in this article. In addition, one more recent paper (10) published after the initial search were added to the discussion of findings, as it appeared relevant for the discussion.

Studies were considered for inclusion if they were written in English or Spanish, published as a full article, if they compared subjects with SZ and BD, or subjects with either SZ or BD with healthy controls (HC), addressing differences between groups.

## 3 Results

### 3.1 Epidemiology and risk factors

The lifetime prevalence of SZ is about 1% (11), although numbers vary with the study design. In addition to a genetic predisposition, there are epigenetic risk factors in SZ such as urban life, poverty, childhood trauma, abandonment and prenatal infections (12–14). The clinical manifestation usually begins in late adolescence, in the majority of cases with residual poor psychosocial function (15). The strong genetic predisposition remains throughout life, and frequent recurrences of psychotic episodes is rather the rule than the exception (16, 17). Historic concordance rates reported for monozygotic twins are around 60% (18); however, a more recent analysis of Danish registries found concordance rates of 33% in monozygotic twins and 7% in dizygotic twins (19). This lower-than-expected concordance rate in monozygotic twins demonstrates that vulnerability to the disease is not solely due to genetic factors. Besides the already mentioned psychosocial adversities, early developmental complications such as maternal exposure to starvation and influenza in the second trimester of pregnancy, birth weight < 2500 g, Rhesus incompatibility during the second pregnancy, and hypoxia during birth additionally increase the risk (20). In addition, childhood trauma and adversities also contribute to the manifestation and severity of schizophrenia (21), according to a meta-analysis the odds ratio (OR) for an association of childhood adversities and psychosis is OR=2,78 (22).

BD comprises a clinical picture characterized by the alternance of depressive, mixed and manic or hypomanic poles. Although a DSM-5 (23) or ICD-11 (24) categorial diagnosis is established by the occurrence of a single manic, hypomanic or mixed episode, depressive episodes are those that carry most of the burden of this chronic and disabling disease. Similar to SZ, the age of onset of BD is usually between 15 and 30 years – the second and third decade of life with some variance between subtypes (25). For BD type I (BD-I), the average age of onset is 18.4 years, for BD type II (BD-II), 20 years, and for subclinical forms, 21.9 years (25). Additionally, a large cohort study also establishes a second peak of onset between the age of 45 and 54 years (26). Variations of age at onset, however, are difficult to identify accurately due to the latency in both the diagnosis and the beginning of treatment (27). However, half of the patients usually have the first episode before the age of 25, and there is a reciprocal relationship between the age of onset and the severity of the BD subtype- the younger the patient at onset of BD, the more severe is the long-term course (28, 29).

A delay of several years is common from the onset of the first affective episode to the diagnosis of BD. The average delay in reaching the correct diagnosis of BD is 8 to 10 years, with the consequence of both a delay in receiving adequate treatment as well as a higher possibility of receiving inappropriate medication that may trigger further episodes (30). Achieving a correct diagnosis and initiating optimal treatment may become even more difficult since more than half of patients with BD have an additional diagnosis, the most common being alcohol or drug abuse disorder (31).

BD is highly heritable (32), reported concordance rates for monozygotic twins range from 40-70%, similar to SZ (33). Again, recent studies show the additional role of several environmental factors. Childhood adversities, ranging from neglect to physical and sexual abuse, have a significant contribution to the manifestation of bipolar disorder in adolescence and early adulthood (34). A meta-analysis of 19 eligible studies showed that childhood adversity was 2.63 times (95% CI 2.00-3.47) more likely to have occurred in subjects with bipolar disorder compared to non-clinical controls (35). In addition, bipolar patients who experienced childhood adversities also tend to have a more detrimental course of the disorder, including a higher risk of suicide attempts and medical comorbidities (36). Although stressful life events in later life are not believed to be the cause of BD per se, they may trigger recurrence. A role of perinatal complications, maternal influenza infection during pregnancy, and maternal exposure to smoking *in utero* and tuberculosis risk is supported by relatively strong evidence, as well as advanced paternal age at conception is a risk factor, suggesting a role for *de novo* mutations (37).

In summary, genetic predisposition plays a dominant role in both disorders (see also next chapter). Perinatal complications and childhood adversities constitute risk factors for the manifestation of both SZ and BD, and have a detrimental effect on the course of illness. Environmental stressors can trigger onset or relapse in individuals vulnerable to both disorders by initiating epigenetic changes (38). Thus, these factors do not differentiate between SZ and BD, and rather appear to be a common feature of severe mental disorders in general.

### 3.2 Genetic data

SZ and BD are strongly determined by inheritance. The contribution of heritability has been estimated up to 70-80% % for both SZ (19) and BD (33). Concordance in twins is around 40% to 45% in monozygotics and 0% to 10% in dizygotics for SZ and BD (39). Significant progress has been made more recently in the discovery of specific genetic loci associated with these disorders. Genome-wide association studies (GWAS) have discovered 270 single nucleotide polymorphisms (SNPs) that confer risk of SZ (40) and 64 for BD (41) with some overlapping associations. The Bipolar Disorder and Schizophrenia Working Group of the Psychiatric Genomics Consortium performed association analysis of BD and SCZ combined into a single phenotype, totaling 53,555 cases (20,129 BD, 33,426 SCZ) and 54,065 controls on 15.5 million SNP allele dosages imputed from 1000 genomes phase 3 (42). They identified 114 genomic risk loci shared between SZ and BD (43). These overlapping associations may constitute the biological substrate of the observed co-aggregation of the two disorders in families (44, 45). On the other hand, many of these common genetic variants are not shared between these disorders and other forms of genetic variation, such as copy number variants (CNVs) (46, 47) and rare variants identified by sequencing (48, 49) which appear to play a much more prominent role in the genetic architecture of SZ than in BD (50).

A meta-analysis published in 2019 of GWAS included 20,352 cases and 31,358 controls of European ancestry, with follow-up of the main findings in an independent sample of 9,412 cases and 137,760 controls. 30 loci associated with bipolar disorder stand out; 8 of these also harbor associations with SZ. Relevant loci contain genes that encode, among other things, ion channels, neurotransmitter transporters and synaptic components. The meta-analysis also demonstrates the genetic overlap of BD-I and SZ, including psychosis, as well as BD-II with major depressive disorder (MDD) (51).

In a study carried out by Akihito Uezato and collaborators, significant allelic and genotypic associations were found between the SLC35B2 gene and BD, as well as a decrease in the expression of SLC35B2 mRNA in the dorso-lateral prefrontal cortex (DLPFC) in bipolar patients. On the other hand, in patients with SZ, no modifications were observed in mRNA expression in DLPFC or anterior cingulate cortex (ACC), although there was a significant genetic association with SZ (52).

Gender differences in onset and course of both SZ and BD may be, at least in part, also genetically determined. GWAS showed significant sex-dependent effects for genes related to neuronal development, immune and vascular functions across and within SCZ, BD, and MDD both at the variant, gene, and pathway levels (53).

As mentioned above, environmental variables have been studied in both disorders; however, fewer studies have been carried out in BD, with smaller samples and with inconsistent results. It is important to highlight that the impact environmental exposure can generate is related to the genetic structure of that individual. This highlights the importance of studying the relationship between genes and environment. For example, the effect of infections during pregnancy and the risk of developing SZ has been well established. Furthermore, there is a genetic correlation between susceptibility to infection and psychiatric disorders in general, reported in a GWAS conducted in a large Danish cohort, revealing shared genetic factors (54). We can further add that the variation of the Major Histocompatibility Complex (MHC) region associated with potential susceptibility to infections (55) has been associated with SZ and recently also with BD (56, 57).

A number of peripheral immune markers have been associated with specific categories of psychopathology such as positive symptomatology and cognitive deficits in both SZ and BD (58, 59). However, the relationship between complement expression and clinical variables in these disorders has not been studied in depth, paying attention not only to the diagnoses but also to the association between immune activation and symptoms. Melbourne and her colleagues demonstrated a positive association between C4A mRNA expression and delusions (60).

Epigenetic alterations (modifications of chromatin structure, measured by assessing the degree of methylation of CpG dinucleotides) are closely related to gene expression, procuring the aging process of cells and affecting their function (61). Research carried out on epigenetic age, individuals with SZ, BD, children at familiar high risk of SZ or BD, and healthy controls delivered inconclusive results. Overall, the accelerated aging hypothesis in schizophrenia could not be confirmed, and clear-cut differences between SZ and BD not be established (38).

Summarizing results from genetic studies, GWAS identified discrete SNPs associated with the risk of SZ or BD, several of them overlapping (43). However, CNVs and rare variants appear to play a more prominent role in SZ, whereas the heritability of BD is largely attributed to multiple small-effect loci. Of the two main manifestations of BD, BD-I displays more genetic similarities with SZ than BD-II. As a future prospect, a Bipolar Schizophrenia Network on Intermediate Phenotypes (B-SNIP) has been created to examine psychotic disorders with a wide variety of intermediate phenotypes and aims to use information from these biomarkers in the search towards a biomarker-based classification of psychoses (62). However, it has to be kept in mind that SNPs may not have any relevant impact in physiological terms, unless the SNPs in question has/have been demonstrated to have an actual cellular effect.

Looking at epigenetic differences, perinatal infections seem to contribute to risk for both disorders, but also other factors such as early childhood adversities, psychosocial stress and trauma are likely to be involved in increased epigenetic risk for both disorders.

### 3.3 Neuroimaging data

Despite advances in the field of neuroimaging, there is no pathognomonic finding so far that differentiates unequivocally BD from SZ, yet some quantitative differences have been described. In a previous review conducted by Grunze and Cetkovich-Bakmas in 2021 some findings were detailed such as hippocampal volume reduction observed in SZ but not in BD, or cortical reductions in the orbitofrontal cortex of BD and in dorsal and frontal temporal areas of SZ patients that also correlated with cognitive deficiencies, among other findings (6).

Ian Ellison-Wright conducted a meta-analysis in 2010 where they compared maps of brain structural abnormalities in 2058 patients with SZ with 2131 healthy controls and 366 patients with BD and 497 healthy controls (HC). Meta-analysis of this data identified four regions of gray matter decreases in BD compared with controls on sum-rank analysis. The regions were in the right insula, perigenual anterior cingulate, left insula and subgenual anterior cingulate. In SZ, meta-analysis of the data identified two regions of gray matter decreases when compared with controls on sum-rank analysis: First, an extensive region covering the insula bilaterally (extending into dorsolateral prefrontal cortex and superior temporal cortex and bilateral hippocampal– amygdala region), thalamus, anterior cingulate and medial-frontal gyrus. The second region was in the posterior cingulate. In addition, meta-analysis of gray matter increase co-ordinates in SZ identified a region extending from the right globus pallidus to the left caudate. For the GM reduction, 72 BD co-ordinates were within the grey matter brain mask of which 27 were inside the regions of GM reduction in SZ. This over-representation was significant (p < 0.01). In summary, the regions of GM reduction in BD were less extensive than the GM reductions in SZ but were substantially overlapping. Only a distinctive region of the pregenual cingulate cortex (anterior Brodmann area 24) stood out where GM reduction was detected in BD studies but not in SZ studies (63).

The Enhancing Neuroimaging Genetics through Meta Analysis Consortium (ENIGMA) conducted the largest magnetic resonance imaging (MRI) studies of patients with SZ and BD so far, reporting that the hippocampus had the largest volume reduction of all subcortical brain structures in both disorders (64, 65). These findings were also supported by K. Haukvik and colleagues who conducted a systematic review and meta-analysis of 21 MRI studies to address and compare hippocampal subfield abnormalities in both disorders (66).

Several functional MRI (fMRI) studies have found abnormalities of the ventral prefrontal cortex and striatal activation in BD whereas reduced dorsal prefrontal cortex activation was found in SZ. To test the hypothesis that brain areas involved in word production can help to differentiate BD from SZ, Andrew McIntosh and colleagues recruited 42 outpatients with BD-I, 27 outpatients with SZ and 37 HC. Compared to SZ and HC, they found differences of fronto-striatal systems associated with performance on a set shifting task (Hayling Sentence Completion Test) in BD patients (67). BD patients engaged emotional brain areas more than HC and SZ patients while performing the Hayling task. These results support the hypothesis that circuits involving the dorsal prefrontal cortex are implicated in the pathophysiology of SZ, whereas circuits involving the lateral orbitofrontal cortex are more specifically implicated in BD.

In 2016 Liberg et al. conducted a narrative review to investigate whether longitudinal brain trajectories of dynamic illness features could help differentiate between SZ and BD. In this review, authors detailed illness-specific trajectories of morphological change in total grey matter volume and in regions of the frontal, temporal and cingulate cortices. Also, a trajectory involving progressive grey matter loss confined to fronto-temporal cortical regions was found in SZ patients. Authors also described this less severely impacted trajectory in a number of regions in bipolar disorders (68). However, as this is a narrative review based on a small number of studies, results may be taken with caution.

Last but not least, BrainAGE (brain age gap estimation) is a novel morphometric parameter using a machine learning approach. The BrainAGE score was developed to estimate the age from individual MRI images based on the reduction of multi-variate age-related grey matter effects across the whole brain (69). The difference between each individual’s estimated and chronological age results in the BrainAGE providing an indication of deviation from normal aging trajectories. Lower scores might indicate developmental delays, a higher BrainAGE score accelerated aging. Using this approach, Nenadić and colleagues conducted a case-control study analyzing MRI data and BrainAge quantification of acceleration or deceleration of individual aging. Data from 45 SZ patients, 22 BD-I and 70 HC was analyzed concluding that SZ patients, but not BD-I patients, had higher rates of BrainAge scores than HC. These findings suggest the presence of an additional progressive pathogenic (neurodegenerative) component in SZ despite the conceptualization of SZ as a neurodevelopmental disorder (70).

In 2022 Zhao et al. conducted a multimodal meta-analysis of anisotropy and volume abnormalities in white matter (WM) comparing patients with SZ, BD and HC. The bilateral corpus callosum showed shared decreased WM volume and fractional anisotropy in both SZ and BD. Compared with BD patients, SZ patients showed remarkable more extensive disorder-specific WM abnormalities: decreased FA and increased WM volume in the left cingulum, and increased FA plus decreased WM volume in the right anterior limb of the internal capsule (71).

In summary, up to date no pathognomonic neuroimaging finding has been identified allowing to make the diagnosis of either SZ or BD. Both GM and WM abnormalities overlap in SZ and BD, but some findings, e.g., GM reduction in the pregenual cingulate cortex in BD, can be related to one or the other supporting the hypothesis of discrete disorders. Brain age gap estimation suggests that SZ is not a purely neurodevelopmental disorder, but has, similar to BD, also a neurodegenerative component.

### 3.4 Postmortem data

In a recent review and meta-analysis, Harrison and colleagues (72) concluded that no neuropathological findings in bipolar disorder can be considered to have been established beyond reasonable doubt due to small sample sizes in singular studies and lack of replication studies. Nevertheless, some replicated findings resemble closely what has been observed *in vivo* in neuroimaging studies, namely decreased cortical thickness and glial density in subgenual anterior cingulate cortex and reduced neuronal density in the lateral, basal and accessory basal nuclei of the amygdala (73, 74). Several post- mortem studies also described decreased calbindin-positive neuron density in the prefrontal cortex. It has been hypothesized from animal studies that calbindin-positive neurons are regulating brain networks involved in spatial navigation, memory processes, and social interactions (75). Main focus of post-mortem research in SZ has been the hippocampal formation, prefrontal cortex thalamus. In line with neuroimaging data, postmortem studies showed a significant reduction of volume and neuron number in multiple hippocampal subfields in left, but not right hippocampus. Neuron size, averaged bilaterally, was also significantly reduced (76). Changes in fast-spiking GABAergic interneurons containing the parvalbumin (PV) are well established in SZ (77), reductions in the number and density of PV interneurons in the prefrontal cortex and hippocampus have been found in the postmortem brain of individuals with SZ (78, 79), and abnormal PV interneuron function in the PFC has been associated with cognitive impairments and negative symptoms (80).For the thalamus, two meta-analyses indicated a small-to-moderate effect size for thalamic size reduction in schizophrenia (81).

Thus, similar to neuroimaging, post-mortem findings suggest a more pronounced role of the hippocampal formation in SZ, whereas changes of the cingulate cortex and amygdala are more pathognomic for BD. However, as both disorders are network affections, it comes with no surprise that there is no single pathognomic brain structure that is solely affected by one, but not the other disorder, and that there are common regions of interest such as the prefrontal cortex in both disorders. Also of note, the vast majority of postmortem examinations found no evidence for excessive gliosis in SZ and BD, making them distinct from neurodegenerative disorders such as Alzheimer`s disease (72, 82).

### 3.5 Cognition: neurodevelopmental vs. neuroprogressive impairment

Both SZ and BD are disorders characterized by cognitive impairment (83–87). In SZ, the degree of neurocognitive dysfunction correlates negatively with the functional outcome (83). With regard to BD, Bortolato and colleagues suggested that there is evidence of cognitive dysfunction across several domains, although less severe compared to SZ. As neurocognitive peculiarities have been observed in both disorders prior to clinical manifestations and also in first degree relatives, they appear to be, at least in part, caused by neurodevelopmental irregularities. This notion has been supported by a recent study by Menkes and colleagues examining a large sample of early and chronic stage schizophrenia and psychotic bipolar patients, reporting evidence for neurodevelopmental and neuroprogressive cognitive trajectories in both disorders (88). The situation is less clear in non-psychotic BD. Results are, in part, contradictory; thus, it is currently unknown whether BD is characterized by cognitive stability or progression after illness onset (Van Rheenen et al., 2020). Contradictory results in cognition research may, in part, originate from patient selection. More recent studies comparing cohorts of bipolar and schizophrenic individuals found that the long- term cognitive outcome does not only rely on the diagnostic category (SZ vs, BD). Within diagnostic groups there is a large heterogeneity of type and extend of cognitive deficits, and premorbid intelligence predicts to a large degree cognitive outcome in later life. Both in SZ and BD, three discrete cognitive subgroups can been clustered: severely impaired, mildly impaired, and relatively intact. The study by Vaskinn and colleagues (89) assessed 223 individuals with SZ, 175 with BDI and 476 HC. Looking at individuals with SZ and BD, cluster analyses consistently yielded those three clusters: the relatively intact group (36% of whole sample), the intermediate group with mild cognitive impairment (44%), and the impaired group with global deficits (20%). Clusters differed significantly for neuropsychological, functional, and clinical outcome measures. Of note, 32% of the SZ sample and 42% of the BD sample belonged to the relatively intact group, supporting the notion that premorbid intellectual functioning is probably a more decisive predictor for outcome than categorial diagnosis. The authors of the study concluded that highlighting a neuropsychological assessment is needed for the precise characterization of the individual independent from diagnosis SZ or BD.

### 3.6 Cognitive dysfunction in SZ and BD

Multiple meta-analyses report deficits of large effect sizes (ES) in a broad range of cognitive domains, such as intellectual function, learning and memory, attention, working memory, language and executive function (90–94).

In recent decades, a substantial amount of data has been collected indicating that cognitive deficits in SZ are to a large extent neurodevelopmental (83, 95–98). Discrete premorbid cognitive dysfunction may suggest deviant development or is a precursor of the disorder that will manifest itself later. In line with this, a meta-analysis of longitudinal population-based studies showed that people with SZ had reduced premorbid IQs, and that this was associated with an earlier onset of the illness (99). Even in early to mid-adolescence, a moderately reduced IQ appears to be an indicator of the imminent manifestation of the illness (100).

Cognitive deficits and impaired adjustment to the environment are prominent features not only at first psychotic episode, but also in individuals at high-risk, even at early stages of infancy (101–103). The severity of these widespread cognitive deficits may get close to that of adults with chronic illness, even being more pronounced in immediate verbal memory and processing speed (104). Similarly, it has been reported that intellectual impairment is comparable to that seen in more advanced phases of the illness, supporting the aforementioned neurodevelopmental hypothesis (83).

However, with respect to the longitudinal course of cognitive deficits, it has been suggested that small-to-moderate improvements in several domains are possible after illness onset (105, 106) with largest ES estimates observed in tasks of memory and attention and also in cognitive flexibility (107).

Several factors have been identified as possible moderators of cognitive performance in this population, such as chronicity, symptomatic severity, comorbidity and medication status and dosage. Moreover, negative symptoms and disorganization seem to be related to deficits in executive functions and intellectual functioning (83). Also, some authors suggest that -in comparison to patients with an adult onset- early onset is associated with greater severity of cognitive deficits, such as larger deficits in IQ, executive functioning, psychomotor speed, and verbal memory (108).

Regarding BD, it has been consistently related to cognitive dysfunction in several cognitive domains (83). There are subtle differences in the extent of cognitive dysfunction between BD-I and BD-II patients, but the overall pattern of cognitive deficits is similar (109). These deficits persist even in periods of euthymia (110, 111) and -as a consequence- have been regarded as trait markers of the disorder. Moreover, it has been suggested that following a first episode of BD, young adults present a generalized cognitive impairment (101) similar to the medium effect that has been reported by studies that included also individuals with recurrent episodes (Bourne, Aydemir, Balanza-Martinez, et al., 2013).

This evidence is consistent with a staging model for the disorder based on neuroprogression, characterized by progressive deterioration over time in BD patients (112, 113). However, the theory of neuroprogression has also been challenged (114). Of interest, contrary to data from subjects with several recurrences of mood episodes (115), and also different from SZ patients with a first psychotic episode, visual memory seems to be not impaired after a first bipolar episode (116).

Psychotic symptoms have been related with worse cognitive functioning in comparison with nonpsychotic BD patients in tasks of verbal memory, planning and reasoning, working memory and processing speed (117).

Regarding differences in cognitive function between individuals with BD type I and BD type II, a meta-analysis found a greater impairment in verbal memory in BD-I, while no differences were observed in global cognition, attention, working memory, and executive function (118). The authors posited that memory impairment might be a specific endophenotype for type I of BD, and may result from the neuroprogressive alterations related with manic episodes.

With respect to the longitudinal course of cognitive deficits, the current evidence still is scant and inconsistent (83). Lewandowski and colleagues (119) have conceptualized BD as a neuroprogressive disease characterized by a subtle neurodevelopmental component that precedes illness onset, and which is followed by neurodegeneration after onset and progressive cognitive decline as a function of recurrences. The presence of a neurodevelopmental component of cognitive dysfunction in BD is supported by studies carried out in pediatric BD populations, showing cognitive deficits across different cognitive domains (120). Cross-sectional studies have proposed an association between cognitive impairment and the cumulative number of episodes (121) as a representation of the neuroprogressive nature of BD (122) although the direction of causality is not understood. Likewise, some studies have not identified any distinctive differences in neurocognitive performance between patients and control (123). It is possible that BD may be a heterogeneous phenotype, which could cause diverse levels of cognitive impairment based on the respective share of genetic and epigenetic contributions (83).

Compared with SZ, there is scarce evidence regarding premorbid global intellectual impairment in BD. In this context, it has been proposed that neurodevelopmental deficits occur in both SZ and BD, but appear to play a more prominent role in SZ. And there are distinctive differences, at least early in the course of illness, such as the extent of visual memory impairment, supporting again the notion of distinctive disorders (85, 103).

Considering both disorders, there is evidence that they have a large symptomatic and etiological overlap, and many authors no longer consider SZ and BD as distinctive or categorical entities (86, 124). The difference in cognition between SZ and BD seems to be more quantitative (degree of deficit) rather than qualitative (profile), supporting a dimensional approach. In this context, it is suggested that SZ patients show more severe and pervasive deficits while BD patients present a milder and more confined impairment (85–87). One meta-analysis indicated that BD patients showed better cognitive performance than SZ patients in measures like verbal fluency, verbal working memory, mental speed, executive control, and immediate verbal memory (125). Another meta-analysis indicated similar results, with SZ patients producing inferior results in 6 out of 12 cognitive domains (126).

The longitudinal course of cognitive impairment also differs between groups: premorbid cognitive impairment is present already early in life in SZ (often childhood/adolescence), preceding the onset of psychosis, but visible cognitive impairment in BD typically does not occur until later in adulthood, after many years of recurrent mood episodes. Meta-analysis of data on initial stages of BD and SZ showed that neuropsychological performance of first-episode manic BD patients was impaired, albeit to a lesser degree than first-episode SZ patients and healthy controls. Notably, those who had a first episode of mania exhibited a level of impairment comparable to that found in those with more chronicity (103, 126).

Zanelli and colleagues (127) suggested that both SZ and bipolar I patients experience cognitive decline in general and specific functions after the first episode, but the age at which cognitive fading occurs differs between disorder and intellectual function. Another study evaluated whether SZ and BD show different patterns of episodic memory (EM) performance, according to their clinical stages (128). The results showed that in SZ, EM impairment occurs in the early stage of illness, whereas in BD patients, the cognitive decline was found alongside with the progression of the illness. In other words, patients with a recent-onset SZ performed similar to chronic patients. In contrast, individuals with early-stage BD and healthy controls, demonstrated similar cognitive capabilities and performed better than late-stage BD group. With regard to this last group, it was found the same degree of EM dysfunction as in individuals with SZ. The authors argued that these findings corroborate the hypothesis of BD as a progressive disease that impairs cognition along its course (129–131).

One study aimed to compare cognitive dysfunction between BD and SZ across symptomatic and remitted states (84). Results showed that both disorders are related to neurocognitive alterations which are state dependent (such as global cognition and working memory) and diagnosis dependent (such as global cognition and verbal fluency). These deficits were more pronounced in SZ. Processing speed was in a normal range in remitted SZ and BD patients, as well as symptomatic BD patients, but significantly slowed down in symptomatic states of schizophrenia. It has been hypothesized that these neuropsychological differences between groups could be due to the presence of psychotic features, to environmental factors (such as stressful events, duration of the disease and number of hospitalizations) and could also be related to differences during the neurodevelopmental phase (85), the latter supporting again the notion of different entities.

### 3.7 Phenomenology

With the emergence of second-generation antipsychotics, ranked highly in treatment algorithms in both SZ and BD, diagnostic diligence appears to have taken a back seat. Differential diagnosis has been reduced to the presence or absence of psychotic symptoms leaving out performance and functional impairment in SZ. In the absence of a unique and easy to capture biological marker, full assessment of the phenomenology is key for a proper diagnosis. The course of illness may be one of the most important criteria when making the differential diagnoses between the two disorders, whereas a pure cross-sectional view of acute symptomatology may direct the physician in the wrong direction (27). Moreover, psychotic symptoms in both BD and SZ have several similarities (27). Regarding positive symptoms, Schneiderian first rank symptoms (FRS) had been suggested as a criterion to differentiate SZ from other psychotic disorders. In 2020, Peralta and Cuesta concluded a study with 1146 subjects addressing the validity of FRS (132). Their findings suggest that not only FRS do not have a diagnostic value for diagnosing SZ nor add validity compared to other delusions and hallucinations to a diagnosis of SZ.

Regarding the thought domain, formal thought disorder has been considered as a possible distinctive symptom of SZ. In 2016, Yalincentin and colleagues conducted a meta-analysis addressing formal thought disorders in BD and SZ patients (133). They compared positive (PTD) and negative (NTD) formal thought disorder in both disorders. Their findings suggested that PTD is a shared feature of both SZ and BP but persistent PTD or NTD can distinguish subgroups of SZ from BP and SZ patients with better clinical outcomes.

Regarding early symptoms of each disease, Andersen and colleagues conducted a retrospective study comparing the type and frequency of diagnoses preceding adult BD and SZ (134). The number of patients with any preceding diagnosis amounted to 69.3% in BD and 76.6% in SZ, and patients with SZ had also minor but statistically significant earlier onset of any psychiatric disorder compared to BD patients (mean age: 22.5 vs 23.3, p<.001). Regression analyses indicated that BD was associated with an increased risk of previously diagnosed affective disorders and Attention-Deficit/Hyperactivity Disorder (ADHD). As a caveat, there is still an intense debate ongoing whether or not ADHD is comorbid to BD or a frequent misdiagnosis of early signs of BD. SZ was associated with an increased risk of preceding substance use disorders, psychosis, anxiety disorders and personality disorders diagnoses. Researchers concluded that different patterns of vulnerability in terms of preceding diagnoses were associated with the two diagnoses, with affective disorders being more specific to BD, and both substance use disorders and psychosis to SZ.

Another important domain to analyze is the affective share in both disorders. Psychotic mood disorders are difficult to differentiate from SZ, mainly because there is a wrong conception of positive symptoms as the core of SZ. Fountoulakis et al. conducted a study with 175 patients with SZ to evaluate the presence of manic symptoms. Significant subthreshold manic symptoms were found in 25.14% patients. Mood symptoms correlated with positive symptoms (135). This study suggests that many patients are misdiagnosed with SZ while they currently suffer from a psychotic affective disorder.

However, also schizophrenia patients frequently manifest mood symptoms. Increased emotional arousal and reactivity is frequently observed together with positive symptoms, termed ‘the emotional paradox’ of schizophrenia (136). Likewise, depression is common in schizophrenia as part of the prodromal and florid phase, following an acute episode (“postpsychotic depression”), or occurring between psychotic exacerbations (137). The prevalence of depressive disorder in schizophrenia has been reported to be around 40%, however the stage (early vs chronic) and state (acute or post-psychotic) influences figures. In acute episodes rates are up to 60%, whilst in post-psychotic schizophrenia rates of moderate to severe depression vary between 20% in chronic schizophrenia and 50% following treatment of first episode (121).

In summary, SZ and BD have several symptoms in common in a variety of domains such as affective or positive symptoms. However, a differential diagnosis can be done by delineating the course of illness, although it can be challenging to analyze a first episode retrospectively in the absence of a medical history supplied by relatives/partner etc.

## 4 Discussion

In this review of the differences between SZ and BD we highlighted findings in epidemiological, genetic, neuroimaging, phenomenological and cognition data. Meanwhile there is good evidence supporting the hypothesis of SZ and BD as discrete disorders, yet some overlapping exists.

There is little evidence pointing towards SZ and BD being part of a spectrum; yet more evidence would be needed to move away from a categorical to a continuum perspective. A main reason that keeps this discussion ongoing may be that in some patients the current diagnostic tools and the current genetic and clinical data do not “work together”. Regarding phenomenology and current diagnostic tools, the main focus in SZ is on positive symptoms while, as we had discussed, these can also be transiently present in BD. As we suggested, the focus should be on the longitudinal course of illness instead.

Unfortunately, neuroimaging cannot resolve diagnostic uncertainty. Even though some neuroimaging studies support subtle differences between disorders, there is no unequivocally unique finding distinguishing SZ from BD.

The coincidence in age of onset in SZ and BD is not necessarily an argument that supports the hypothesis of a single disorder; in fact, it is also the common age of onset of several severe mental disorders while the brain is still maturating. The evidence for heritability of both SZ and BD is obvious, as well as the environmental impact on individual manifestations of both disorders. Environmental stressors can trigger onset and relapse by initiating epigenetic changes in both disorders. So far, a genetic correlation has been observed between BD type I and SZ, including psychosis on the one hand, and BD type II and depression on the other. And although SNPs associated with both SZ and BD were identified, CNVs and rare variants seem to have a more relevant role in SZ, while multiple loci with small effects in BD.

A downside of genetic research in BD and SZ is the fact that the source of information are isolated peripheral cells, not mature human brain cells with their differentiation (neurons, interneurons, astrocytes etc.) interacting with each other. GWAS and CNV association studies have reproducibly identified numerous risk alleles associated with BD and SZ but biological characterization of these alleles lags gene discovery, owing to the inaccessibility of live human brain cells. Olfactory Neuroepithelial Cells (138) or Monocyte-Derived-Neuronal-like Cells (139) may be suitable future models, but, again, are distinct from brain tissues and do not resemble fully brain neurons and the complexity of a neuronal network. A more promising approach may be Human-derived induced pluripotent stem cells (iPSCs) that can be differentiated into living, disease-relevant cells and 3D brain organoids carrying the full complement of genetic variants present in the donor germline. Re-programming methods enable generation of iPSCs from patient fibroblasts and peripheral blood mononuclear cells has opened possibilities for new approaches to study relevant disease biology using iPSC-derived neurons. The use of iPSC-derived cells may allow functional characterization of risk alleles, understanding of causal relationships between genes and neurobiology, and screening for potential targets of therapeutics (10, 140, 141). First results showing deviations in gene expression in iPSCs from individuals with BD and SZ compared to HC have been published, but samples are still small and direct comparison studies between BD and SZ are still lacking (141). Thus, these studies are not yet helpful to distinguish between BD and SZ, but may be in the future with more data collected.

Looking at the neurobiological basis of BD and SZ, we notice that aberrations in several downstream neurotransmitters, namely the biogenic amines, GABA and glutamate are related to the different phases of both disorders, and their correction is a therapeutic target, although causality and specificity to one or the other disorder remains vague. Inflammation also appears to play a role in the progression of both disorders. The immune-inflammation response is an essential component of the multifactorial disease pathogenesis in major psychiatric disorders. Whereas most investigated inflammation markers are not distinct between major psychiatric illnesses, a recent finding seems to separate SZ, BD (manic phase) and BD (depressive phase). In a large sample of individuals with SZ or BD, Wei and colleagues found a relationship between the inflammation and lipid metabolism with differential association patterns in SZ, BD manic, and BD depressed. Thus, the data suggest that specific patterns of inflammation and lipid metabolism markers could be potential biomarkers separating again SZ from BD and may allow for differential diagnosis (142).

Regarding the cognitive profile of SZ and BD, though there is evidence of overlap between these disorders, there are also differences in certain domains, such as premorbid intellectual functioning and executive functions. Moreover, the timing and trajectory of cognitive impairment in both disorders also differs. Deficits tend to appear earlier in SZ, and are more pronounced than in BD. More research data, however, is needed to understand the timing and trajectory of cognitive impairment in both disorders, which in turn might help provide effective treatment options targeting cognition.

Finally, it is worth mentioning that besides several symptom- orientated medication such as antipsychotics, there is at least one medication that is specifically helpful in BD but not in SZ: lithium. Using new techniques as iPSCs to completely elaborate lithium’s mode of action may supply further neurobiological evidence for distinct disorders.

Bipolar disorder and schizophrenia have been studied as separate disorders for 130 years by thousands of psychiatrists throughout the world. It has always been evident that there is a grey area where it remains difficult to establish a firm diagnosis and treatment plan. Neurobiological research’s latest findings may have failed to find unequivocal boundaries between both disorders so far, but, by no account, proved that they are part of a continuum.

## 5 Limitations

Despite the detailed search, this article is not a full and complete review of all available studies on the topic. Although following systematic search criteria, we did not fill out a PRISMA sheet as this review does not claim to be a comprehensive meta-review but a narrative review of key papers that, according to our judgement, make a contribution to the question whether bipolar and schizophrenia are part of the same spectrum or distinctive (and distinguishable) disorders.

The search and selection of papers was also limited to articles in English and Spanish. Selection of papers and conclusions may be biased by the personal view and clinical experience of the authors.

Both bipolar disorder and schizophrenia are dynamic disorders. Whereas comparison of the symptomatology refers almost exclusively to acute mania or acute psychosis with positive symptoms, data of other domains such as cognition are usually gathered in non-acute states. Other characteristics, e.g. genetics and epigenetics, are researched and collected in all phases of the illness but especially epigenetics may change over time and depend on illness trajectories. Thus, proximity and demarcation of BD and SZ is not static but may differ both between individuals and over life span.

Notwithstanding these limitations, the findings from this review may contribute to a better understanding of SZ and BD as distinctive disorders.

## 6 Conclusions

The question whether SZ and BD belong to a continuum or are separate disorders remains a challenge for further research. Overlapping symptoms and neurobiological findings can be explained by the fact that SZ and BD affect the same organ, the brain. To epitomize the dilemma: Liver failure can be caused both by toxic agents (alcohol) and virus infection (hepatitis), but the longitudinal course of symptomatology and treatment differs despite the same terminal outcome. Thus, liver cirrhosis caused by alcohol and by infection are related with overlapping symptoms and potentially fatal outcome, but still are different diseases needing different treatment approaches. Currently, our research tools may not be precise enough to carve out distinctive, unique and irrefutable differences between SZ and BD. Given that differences are subtle, a way to overcome diagnostic uncertainties in the future could be the application of artificial intelligence based on BigData, also in the field of Mental Health. This may support clinicians in making an early and correct diagnosis, avoiding delays in treatment and resulting in better outcomes, both in SZ and BD.

## Author contributions

MD: Writing – original draft. MK: Writing – original draft. DA: Writing – original draft. MC: Writing – review & editing, Supervision, Conceptualization. CB: Writing – review & editing. HG: Writing – review & editing, Conceptualization.

## References

[1] Angst J. Historical aspects of the dichotomy between manic-depressive disorders and schizophrenia. Schizophr Res. (2002) 57:5–13. doi: 10.1016/s0920-9964(02)00328-6 12165371

[2] Angst J Gamma A. A new bipolar spectrum concept: a brief review. Bipolar Disord. (2002) 4 Suppl 1:11–4. doi: 10.1034/j.1399-5618.4.s1.1.x 12479669

[3] van Os J Kapur S. Schizophrenia. Lancet. (2009) 374:635–45. doi: 10.1016/S0140-6736(09)60995-8 19700006

[4] Jablensky A. Prototypes, syndromes and dimensions of psychopathology: an open agenda for research. World Psychiatry. (2012) 11:22–3. doi: 10.1016/j.wpsyc.2012.01.020 PMC3266758 22294999

[5] Beer MD. Psychosis: a history of the concept. Compr Psychiatry. (1996) 37:273–91. doi: 10.1016/s0010-440x(96)90007-3 8826692

[6] Grunze H Cetkovich-Bakmas M. “Apples and pears are similar, but still different things.” Bipolar disorder and schizophrenia- discrete disorders or just dimensions? J Affect Disord. (2021) 290:178–87. doi: 10.1016/j.jad.2021.04.064 34000571

[7] Foucher JR Gawlik M Roth JN de Crespin de Billy C Jeanjean LC Obrecht A. Wernicke-Kleist-Leonhard phenotypes of endogenous psychoses: a review of their validity. Dialogues Clin Neurosci. (2020) 22:37–49. doi: 10.31887/DCNS.2020.22.1/jfoucher 32699504 PMC7365293

[8] Cuesta MJ Sánchez-Torres AM García de Jalón E Moreno-Izco L Gil-Berrozpe GJ Zarzuela A. Empirical validity of Leonhard’s psychoses: A long-term follow-up study of first-episode psychosis patients. Schizophr Res. (2024) 263:237–45. doi: 10.1016/j.schres.2022.12.022 36682995

[9] Bigdeli TB Voloudakis G Barr PB Gorman BR Genovese G Peterson RE. Penetrance and pleiotropy of polygenic risk scores for schizophrenia, bipolar disorder, and depression among adults in the US veterans affairs health care system. JAMA Psychiatry. (2022) 79:1092–101. doi: 10.1001/jamapsychiatry.2022.2742 PMC9475441 36103194

[10] Detera-Wadleigh SD Kassem L Besancon E Lopes F Akula N Sung H. A resource of induced pluripotent stem cell (iPSC) lines including clinical, genomic, and cellular data from genetically isolated families with mood and psychotic disorders. Transl Psychiatry. (2023) 13:397. doi: 10.1038/s41398-023-02641-w 38104115 PMC10725500

[11] Ruderfer DM Fanous AH Ripke S McQuillin A Amdur RL Gejman PV. Polygenic dissection of diagnosis and clinical dimensions of bipolar disorder and schizophrenia. Mol Psychiatry. (2014) 19:1017–24. doi: 10.1038/mp.2013.138 PMC4033708 24280982

[12] Krabbendam L van Os J. Schizophrenia and urbanicity: a major environmental influence–conditional on genetic risk. Schizophr Bull. (2005) 31:795–9. doi: 10.1093/schbul/sbi060 16150958

[13] McGrath J Saha S Chant D Welham J. Schizophrenia: a concise overview of incidence, prevalence, and mortality. Epidemiol Rev. (2008) 30:67–76. doi: 10.1093/epirev/mxn001 18480098

[14] Stilo SA Murray RM. Non-genetic factors in schizophrenia. Curr Psychiatry Rep. (2019) 21:100. doi: 10.1007/s11920-019-1091-3 31522306 PMC6745031

[15] Dziwota E Stepulak MZ Włoszczak-Szubzda A Olajossy M. Social functioning and the quality of life of patients diagnosed with schizophrenia. Ann Agric Environ Med. (2018) 25:50–5. doi: 10.5604/12321966.1233566 29575877

[16] Haro JM Novick D Suarez D Ochoa S Roca M. Predictors of the course of illness in outpatients with schizophrenia: a prospective three year study. Prog Neuropsychopharmacol Biol Psychiatry. (2008) 32:1287–92. doi: 10.1016/j.pnpbp.2008.04.003 18502012

[17] Tandon R Nasrallah HA Keshavan MS. Schizophrenia, “just the facts” 4. Clinical features and conceptualization. Schizophr Res. (2009) 110:1–23. doi: 10.1016/j.schres.2009.03.005 19328655

[18] Cardno AG Gottesman II. Twin studies of schizophrenia: from bow-and-arrow concordances to star wars Mx and functional genomics. Am J Med Genet. (2000) 97:12–7. 10813800

[19] Hilker R Helenius D Fagerlund B Skytthe A Christensen K Werge TM. Heritability of schizophrenia and schizophrenia spectrum based on the nationwide danish twin register. Biol Psychiatry. (2018) 83:492–8. doi: 10.1016/j.biopsych.2017.08.017 28987712

[20] Hany M Rehman B Azhar Y Chapman J. Schizophrenia. In: StatPearls. StatPearls Publishing LLC, Treasure Island (FL (2023).

[21] Loewy RL Corey S Amirfathi F Dabit S Fulford D Pearson R. Childhood trauma and clinical high risk for psychosis. Schizophr Res. (2019) 205:10–4. doi: 10.1016/j.schres.2018.05.003 PMC6939986 29779964

[22] Varese F Smeets F Drukker M Lieverse R Lataster T Viechtbauer W. Childhood adversities increase the risk of psychosis: a meta-analysis of patient-control, prospective- and cross-sectional cohort studies. Schizophr Bull. (2012) 38:661–71. doi: 10.1093/schbul/sbs050 PMC3406538 22461484

[23] American Psychiatric Association. Diagnostic and statistical manual of mental disorders. 5th. Washington, D.C: APA Press (2013). doi: 10.1176/appi.books.9780890425596

[24] World Health Organisation. ICD-11: International classification of diseases (11th revision) (2022). Available online at: https://icd.who.int/.

[25] Merikangas KR Jin R He JP Kessler RC Lee S Sampson NA. Prevalence and correlates of bipolar spectrum disorder in the world mental health survey initiative. Arch Gen Psychiatry. (2011) 68:241–51. doi: 10.1001/archgenpsychiatry.2011.12 PMC3486639 21383262

[26] Kroon JS Wohlfarth TD Dieleman J Sutterland AL Storosum JG Denys D. Incidence rates and risk factors of bipolar disorder in the general population: a population-based cohort study. Bipolar Disord. (2013) 15:306–13. doi: 10.1111/bdi.12058 23531096

[27] Cetkovich-Bakmas M Abadi A Camino S García Bonetto G Herbst L Marengo E. Tercer Consenso Argentino sobre el manejo de los Trastornos Bipolares. Primera Parte: introducción, método de trabajo y generalidades. Vertex. (2022) 33:56–88. doi: 10.53680/vertex.v33i158.319 36626605

[28] Perlis RH Miyahara S Marangell LB Wisniewski SR Ostacher M DelBello MP. Long-term implications of early onset in bipolar disorder: data from the first 1000 participants in the systematic treatment enhancement program for bipolar disorder (STEP-BD). Biol Psychiatry. (2004) 55:875–81. doi: 10.1016/j.biopsych.2004.01.022 15110730

[29] Post RM Leverich GS Kupka RW Keck PE Jr. McElroy SL Altshuler LL. Early-onset bipolar disorder and treatment delay are risk factors for poor outcome in adulthood. J ClinPsychiatry. (2010) 71:864–72. doi: 10.4088/JCP.08m04994yel 20667291

[30] Vázquez G. Los trastornos bipolares hoy: más allá del DSM–5. In: Psicodebate 14(2) - Diciembre 2014 - Mayo 2015. Facultad de Ciencias Sociales, Universidad de Palermo (2014). p. 9–24.

[31] Grunze H Soyka M. The pharmacotherapeutic management of co-morbid bipolar disorder and alcohol use disorder. Expert Opin Pharmacother. (2022) 23:1181–93. doi: 10.1080/14656566.2022.2083500 35640575

[32] Harrison PJ. Molecular neurobiological clues to the pathogenesis of bipolar disorder. Curr Opin Neurobiol. (2016) 36:1–6. doi: 10.1016/j.conb.2015.07.002 26210959 PMC4779149

[33] Craddock N Sklar P. Genetics of bipolar disorder. Lancet. (2013) 381:1654–62. doi: 10.1016/s0140-6736(13)60855-7 23663951

[34] Post RM Altshuler LL Kupka R McElroy SL Frye MA Rowe M. Verbal abuse, like physical and sexual abuse, in childhood is associated with an earlier onset and more difficult course of bipolar disorder. Bipolar Disord. (2015) 17:323–30. doi: 10.1111/bdi.12268 25307301

[35] Palmier-Claus JE Berry K Bucci S Mansell W Varese F. Relationship between childhood adversity and bipolar affective disorder: systematic review and meta-analysis. Br J Psychiatry. (2016) 209:454–9. doi: 10.1192/bjp.bp.115.179655 27758835

[36] Leverich GS Altshuler LL Frye MA Suppes T Keck PE McElroy SL. Factors associated with suicide attempts in 648 patients with bipolar disorder in the stanley foundation bipolar network. J Clin Psychiatry. (2003) 64:506–15. doi: 10.4088/JCP.v64n0503 12755652

[37] Kato T. Current understanding of bipolar disorder: Toward integration of biological basis and treatment strategies. Psychiatry Clin Neurosci. (2019) 73:526–40. doi: 10.1111/pcn.12852 31021488

[38] Segura AG de la Serna E Sugranyes G Baeza I Valli I Díaz-Caneja C. Epigenetic age deacceleration in youth at familial risk for schizophrenia and bipolar disorder. Transl Psychiatry. (2023) 13:155. doi: 10.1038/s41398-023-02463-w 37156786 PMC10167217

[39] Cardno AG Owen MJ. Genetic relationships between schizophrenia, bipolar disorder, and schizoaffective disorder. Schizophr Bull. (2014) 40:504–15. doi: 10.1093/schbul/sbu016 PMC3984527 24567502

[40] Trubetskoy V Pardiñas AF Qi T Panagiotaropoulou G Awasthi S Bigdeli TB. Mapping genomic loci implicates genes and synaptic biology in schizophrenia. Nature. (2022) 604:502–8. doi: 10.1038/s41586-022-04434-5 PMC9392466 35396580

[41] Mullins N Forstner AJ O’Connell KS Coombes B Coleman JRI Qiao Z. Genome-wide association study of more than 40,000 bipolar disorder cases provides new insights into the underlying biology. Nat Genet. (2021) 53:817–29. doi: 10.1038/s41588-021-00857-4 PMC8192451 34002096

[42] Auton A Brooks LD Durbin RM Garrison EP Kang HM Korbel JO. A global reference for human genetic variation. Nature. (2015) 526:68–74. doi: 10.1038/nature15393 26432245 PMC4750478

[43] Bipolar Disorder and Schizophrenia Working Group of the Psychiatric Genomics Consortium. Genomic dissection of bipolar disorder and schizophrenia, including 28 subphenotypes. Cell. (2018) 173:1705–15.e16. doi: 10.1016/j.cell.2018.05.046 29906448 PMC6432650

[44] Van Snellenberg JX de Candia T. Meta-analytic evidence for familial coaggregation of schizophrenia and bipolar disorder. Arch Gen Psychiatry. (2009) 66:748–55. doi: 10.1001/archgenpsychiatry.2009.64 19581566

[45] Lichtenstein P Yip BH Bjork C Pawitan Y Cannon TD Sullivan PF. Common genetic determinants of schizophrenia and bipolar disorder in Swedish families: a population-based study. Lancet. (2009) 373:234–9. doi: 10.1016/S0140-6736(09)60072-6 PMC3879718 19150704

[46] Malhotra D Sebat J. CNVs: harbingers of a rare variant revolution in psychiatric genetics. Cell. (2012) 148:1223–41. doi: 10.1016/j.cell.2012.02.039 PMC3351385 22424231

[47] Marshall CR Howrigan DP Merico D Thiruvahindrapuram B Wu W Greer DS. Contribution of copy number variants to schizophrenia from a genome-wide study of 41,321 subjects. Nat Genet. (2017) 49:27–35. doi: 10.1038/ng.3725 27869829 PMC5737772

[48] Purcell SM Moran JL Fromer M Ruderfer D Solovieff N Roussos P. A polygenic burden of rare disruptive mutations in schizophrenia. Nature. (2014) 506:185–90. doi: 10.1038/nature12975 PMC4136494 24463508

[49] Goes FS Pirooznia M Parla JS Kramer M Ghiban E Mavruk S. Exome sequencing of familial bipolar disorder. JAMA Psychiatry. (2016) 73:590–7. doi: 10.1001/jamapsychiatry.2016.0251 PMC5600716 27120077

[50] Robinson N Bergen SE. Environmental risk factors for schizophrenia and bipolar disorder and their relationship to genetic risk: current knowledge and future directions. Front Genet. (2021) 12:686666. doi: 10.3389/fgene.2021.686666 34262598 PMC8273311

[51] Stahl EA Breen G Forstner AJ McQuillin A Ripke S Trubetskoy V. Genome-wide association study identifies 30 loci associated with bipolar disorder. Nat Genet. (2019) 51:793–803. doi: 10.1038/s41588-019-0397-8 31043756 PMC6956732

[52] Uezato A Jitoku D Shimazu D Yamamoto N Kurumaji A Iwayama Y. Differential genetic associations and expression of PAPST1/SLC35B2 in bipolar disorder and schizophrenia. J Neural Transm (Vienna). (2022) 129:913–24. doi: 10.1007/s00702-022-02503-7 35501530

[53] Blokland GAM Grove J Chen CY Cotsapas C Tobet S Handa R. Sex-dependent shared and nonshared genetic architecture across mood and psychotic disorders. Biol Psychiatry. (2022) 91:102–17. doi: 10.1016/j.biopsych.2021.02.972 PMC8458480 34099189

[54] Nudel R Benros ME Krebs MD Allesøe RL Lemvigh CK Bybjerg-Grauholm J. Immunity and mental illness: findings from a Danish population-based immunogenetic study of seven psychiatric and neurodevelopmental disorders. Eur J Hum Genet. (2019) 27:1445–55. doi: 10.1038/s41431-019-0402-9 PMC6777475 30976114

[55] Matzaraki V Kumar V Wijmenga C Zhernakova A. The MHC locus and genetic susceptibility to autoimmune and infectious diseases. Genome Biol. (2017) 18:76. doi: 10.1186/s13059-017-1207-1 28449694 PMC5406920

[56] Sekar A Bialas AR de Rivera H Davis A Hammond TR Kamitaki N. Schizophrenia risk from complex variation of complement component 4. Nature. (2016) 530:177–83. doi: 10.1038/nature16549 PMC4752392 26814963

[57] Kamitaki N Sekar A Handsaker RE de Rivera H Tooley K Morris DL. Complement genes contribute sex-biased vulnerability in diverse disorders. Nature. (2020) 582:577–81. doi: 10.1038/s41586-020-2277-x PMC7319891 32499649

[58] Penadés R García-Rizo C Bioque M González-Rodríguez A Cabrera B Mezquida G. The search for new biomarkers for cognition in schizophrenia. Schizophr Res Cognit. (2015) 2:172–8. doi: 10.1016/j.scog.2015.10.004 PMC5609637 29114461

[59] Chase KA Cone JJ Rosen C Sharma RP. The value of interleukin 6 as a peripheral diagnostic marker in schizophrenia. BMC Psychiatry. (2016) 16:152. doi: 10.1186/s12888-016-0866-x 27206977 PMC4874006

[60] Melbourne JK Rosen C Feiner B Sharma RP. C4A mRNA expression in PBMCs predicts the presence and severity of delusions in schizophrenia and bipolar disorder with psychosis. Schizophr Res. (2018) 197:321–7. doi: 10.1016/j.schres.2018.01.018 PMC6087677 29449061

[61] Brunet A Rando TA. Interaction between epigenetic and metabolism in aging stem cells. Curr Opin Cell Biol. (2017) 45:1–7. doi: 10.1016/j.ceb.2016.12.009 28129586 PMC5482778

[62] Tamminga CA Pearlson G Keshavan M Sweeney J Clementz B Thaker G. Bipolar and schizophrenia network for intermediate phenotypes: outcomes across the psychosis continuum. Schizophr Bull. (2014) 40 Suppl 2:S131–7. doi: 10.1093/schbul/sbt179 PMC3934403 24562492

[63] Ellison-Wright I Bullmore E. Anatomy of bipolar disorder and schizophrenia: a meta-analysis. Schizophr Res. (2010) 117:1–12. doi: 10.1016/j.schres.2009.12.022 20071149

[64] Okada N Fukunaga M Yamashita F Koshiyama D Yamamori H Ohi K. Abnormal asymmetries in subcortical brain volume in schizophrenia. Mol Psychiatry. (2016) 21:1460–6. doi: 10.1038/mp.2015.209 PMC5030462 26782053

[65] van Erp TG Hibar DP Rasmussen JM Glahn DC Pearlson GD Andreassen OA. Subcortical brain volume abnormalities in 2028 individuals with schizophrenia and 2540 healthy controls via the ENIGMA consortium. Mol Psychiatry. (2016) 21:547–53. doi: 10.1038/mp.2015.63 PMC4668237 26033243

[66] Haukvik UK Tamnes CK Söderman E Agartz I. Neuroimaging hippocampal subfields in schizophrenia and bipolar disorder: A systematic review and meta-analysis. J Psychiatr Res. (2018) 104:217–26. doi: 10.1016/j.jpsychires.2018.08.012 30107268

[67] McIntosh AM Whalley HC McKirdy J Hall J Sussmann JE Shankar P. Prefrontal function and activation in bipolar disorder and schizophrenia. Am J Psychiatry. (2008) 165:378–84. doi: 10.1176/appi.ajp.2007.07020365 18198268

[68] Liberg B Rahm C Panayiotou A Pantelis C. Brain change trajectories that differentiate the major psychoses. Eur J Clin Invest. (2016) 46:658–74. doi: 10.1111/eci.12641 27208657

[69] Franke K Ziegler G Klöppel S Gaser C. Estimating the age of healthy subjects from T1-weighted MRI scans using kernel methods: exploring the influence of various parameters. Neuroimage. (2010) 50:883–92. doi: 10.1016/j.neuroimage.2010.01.005 20070949

[70] Nenadić I Dietzek M Langbein K Sauer H Gaser C. BrainAGE score indicates accelerated brain aging in schizophrenia, but not bipolar disorder. Psychiatry Res Neuroimaging. (2017) 266:86–9. doi: 10.1016/j.pscychresns.2017.05.006 28628780

[71] Zhao G Lau WKW Wang C Yan H Zhang C Lin K. A comparative multimodal meta-analysis of anisotropy and volume abnormalities in white matter in people suffering from bipolar disorder or schizophrenia. Schizophr Bull. (2022) 48:69–79. doi: 10.1093/schbul/sbab093 34374427 PMC8781378

[72] Harrison PJ Colbourne L Harrison CH. The neuropathology of bipolar disorder: systematic review and meta-analysis. Mol Psychiatry. (2020) 25:1787–808. doi: 10.1038/s41380-018-0213-3 PMC6292507 30127470

[73] Hibar DP Westlye LT Doan NT Jahanshad N Cheung JW Ching CRK. Cortical abnormalities in bipolar disorder: an MRI analysis of 6503 individuals from the ENIGMA Bipolar Disorder Working Group. Mol Psychiatry. (2018) 23:932–42. doi: 10.1038/mp.2017.73 PMC5668195 28461699

[74] Hibar DP Westlye LT van Erp TG Rasmussen J Leonardo CD Faskowitz J. Subcortical volumetric abnormalities in bipolar disorder. Mol Psychiatry. (2016) 21:1710–6. doi: 10.1038/mp.2015.227 PMC5116479 26857596

[75] Bjerke IE Yates SC Laja A Witter MP PuChades MA Bjaalie JG. Densities and numbers of calbindin and parvalbumin positive neurons across the rat and mouse brain. iScience. (2021) 24:101906. doi: 10.1016/j.isci.2020.101906 33385111 PMC7770605

[76] Roeske MJ Konradi C Heckers S Lewis AS. Hippocampal volume and hippocampal neuron density, number and size in schizophrenia: a systematic review and meta-analysis of postmortem studies. Mol Psychiatry. (2021) 26:3524–35. doi: 10.1038/s41380-020-0853-y PMC7854798 32724199

[77] Gonzalez-Burgos G Cho RY Lewis DA. Alterations in cortical network oscillations and parvalbumin neurons in schizophrenia. Biol Psychiatry. (2015) 77:1031–40. doi: 10.1016/j.biopsych.2015.03.010 PMC4444373 25863358

[78] Kaar SJ Angelescu I Marques TR Howes OD. Pre-frontal parvalbumin interneurons in schizophrenia: a meta-analysis of post-mortem studies. J Neural Transm (Vienna). (2019) 126:1637–51. doi: 10.1007/s00702-019-02080-2 PMC6856257 31529297

[79] Konradi C Yang CK Zimmerman EI Lohmann KM Gresch P Pantazopoulos H. Hippocampal interneurons are abnormal in schizophrenia. Schizophr Res. (2011) 131:165–73. doi: 10.1016/j.schres.2011.06.007 PMC3159834 21745723

[80] Knight S McCutcheon R Dwir D Grace AA O’Daly O McGuire P. Hippocampal circuit dysfunction in psychosis. Transl Psychiatry. (2022) 12:344. doi: 10.1038/s41398-022-02115-5 36008395 PMC9411597

[81] Konick LC Friedman L. Meta-analysis of thalamic size in schizophrenia. Biol Psychiatry. (2001) 49:28–38. doi: 10.1016/s0006-3223(00)00974-4 11163777

[82] Harrison PJ. Postmortem studies in schizophrenia. Dialogues Clin Neurosci. (2000) 2:349–57. doi: 10.31887/DCNS.2000.2.4/pharrison PMC3181616 22033474

[83] Bortolato B Miskowiak KW Köhler CA Vieta E Carvalho AF. Cognitive dysfunction in bipolar disorder and schizophrenia: a systematic review of meta-analyses. Neuropsychiatr Dis Treat. (2015) 11:3111–25. doi: 10.2147/NDT PMC4689290 26719696

[84] Ceylan D Akdede BB Bora E Aktener AY Hıdıroğlu Ongun C Tunca Z. Neurocognitive functioning during symptomatic states and remission in bipolar disorder and schizophrenia: A comparative study. Psychiatry Res. (2020) 292:113292. doi: 10.1016/j.psychres.2020.113292 32707217

[85] Daban C Colom F Sanchez-Moreno J Garcia-Amador M Vieta E. Clinical correlates of first-episode polarity in bipolar disorder. ComprPsychiatry. (2006) 47:433–7. doi: 10.1016/j.comppsych.2006.03.009 17067865

[86] Vöhringer PA Barroilhet SA Amerio A Reale ML Alvear K Vergne D. Cognitive impairment in bipolar disorder and schizophrenia: a systematic review. Front Psychiatry. (2013) 4:87. doi: 10.3389/fpsyt.2013.00087 23964248 PMC3737461

[87] Parellada M Gomez-Vallejo S Burdeus M Arango C. Developmental differences between schizophrenia and bipolar disorder. Schizophr Bull. (2017) 43:1176–89. doi: 10.1093/schbul/sbx126 PMC5737496 29045744

[88] Menkes MW Armstrong K Blackford JU Heckers S Woodward ND. Neuropsychological functioning in early and chronic stages of schizophrenia and psychotic bipolar disorder. Schizophr Res. (2019) 206:413–9. doi: 10.1016/j.schres.2018.10.009 PMC6530584 31104720

[89] Vaskinn A Haatveit B Melle I Andreassen OA Ueland T Sundet K. Cognitive heterogeneity across schizophrenia and bipolar disorder: A cluster analysis of intellectual trajectories. J Int Neuropsychol Soc. (2020) 26:860–72. doi: 10.1017/s1355617720000442 32423506

[90] Johnson-Selfridge M Zalewski C. Moderator variables of executive functioning in schizophrenia: meta-analytic findings. Schizophr Bull. (2001) 27:305–16. doi: 10.1093/oxfordjournals.schbul.a006876 11354597

[91] Pelletier M Achim AM Montoya A Lal S Lepage M. Cognitive and clinical moderators of recognition memory in schizophrenia: a meta-analysis. Schizophr Res. (2005) 74:233–52. doi: 10.1016/j.schres.2004.08.017 15722003

[92] Forbes NF Carrick LA McIntosh AM Lawrie SM. Working memory in schizophrenia: a meta-analysis. Psychol Med. (2009) 39:889–905. doi: 10.1017/s0033291708004558 18945379

[93] Wang Y Cui J Chan RC Deng Y Shi H Hong X. Meta-analysis of prospective memory in schizophrenia: nature, extent, and correlates. Schizophr Res. (2009) 114:64–70. doi: 10.1016/j.schres.2009.07.009 19713081

[94] Fioravanti M Bianchi V Cinti ME. Cognitive deficits in schizophrenia: an updated metanalysis of the scientific evidence. BMC Psychiatry. (2012) 12:64. doi: 10.1186/1471-244x-12-64 22715980 PMC3528440

[95] Weinberger DR. Implications of normal brain development for the pathogenesis of schizophrenia. Arch Gen Psychiatry. (1987) 44:660–9. doi: 10.1001/archpsyc.1987.01800190080012 3606332

[96] Murray RM Lewis SW. Is schizophrenia a neurodevelopmental disorder? Br Med J (Clin Res Ed). (1988) 296:63. doi: 10.1136/bmj.296.6614.63 PMC2544668 3122933

[97] Murray RM Bhavsar V Tripoli G Howes O. 30 years on: how the neurodevelopmental hypothesis of schizophrenia morphed into the developmental risk factor model of psychosis. Schizophr Bull. (2017) 43:1190–6. doi: 10.1093/schbul/sbx121 PMC5737804 28981842

[98] Weinberger DR. Future of days past: neurodevelopment and schizophrenia. Schizophr Bull. (2017) 43:1164–8. doi: 10.1093/schbul/sbx118 PMC5737209 29040792

[99] Khandaker GM Barnett JH White IR Jones PB. A quantitative meta-analysis of population-based studies of premorbid intelligence and schizophrenia. Schizophr Res. (2011) 132:220–7. doi: 10.1016/j.schres.2011.06.017 PMC3485562 21764562

[100] Dickson H Laurens KR Cullen AE Hodgins S. Meta-analyses of cognitive and motor function in youth aged 16 years and younger who subsequently develop schizophrenia. Psychol Med. (2012) 42:743–55. doi: 10.1017/s0033291711001693 21896236

[101] Lee RS Hermens DF Scott J Redoblado-Hodge MA Naismith SL Lagopoulos J. A meta-analysis of neuropsychological functioning in first-episode bipolar disorders. J Psychiatr Res. (2014) 57:1–11. doi: 10.1016/j.jpsychires.2014.06.019 25016347

[102] Bora E Pantelis C. Meta-analysis of cognitive impairment in first-episode bipolar disorder: comparison with first-episode schizophrenia and healthy controls. Schizophr Bull. (2015) 41:1095–104. doi: 10.1093/schbul/sbu198 PMC4535631 25616505

[103] Trotta A Murray RM MacCabe JH. Do premorbid and post-onset cognitive functioning differ between schizophrenia and bipolar disorder? A systematic review and meta-analysis. Psychol Med. (2015) 45:381–94. doi: 10.1017/s0033291714001512 25065268

[104] Mesholam-Gately RI Giuliano AJ Goff KP Faraone SV Seidman LJ. Neurocognition in first-episode schizophrenia: a meta-analytic review. Neuropsychology. (2009) 23:315–36. doi: 10.1037/a0014708 19413446

[105] Keefe RS Silva SG Perkins DO Lieberman JA. The effects of atypical antipsychotic drugs on neurocognitive impairment in schizophrenia: a review and meta-analysis. Schizophr Bull. (1999) 25:201–22. doi: 10.1093/oxfordjournals.schbul.a033374 10416727

[106] Harvey PD Keefe RS. Studies of cognitive change in patients with schizophrenia following novel antipsychotic treatment. Am J Psychiatry. (2001) 158:176–84. doi: 10.1176/appi.ajp.158.2.176 11156796

[107] Szöke A Trandafir A Dupont ME Méary A Schürhoff F Leboyer M. Longitudinal studies of cognition in schizophrenia: meta-analysis. Br J Psychiatry. (2008) 192:248–57. doi: 10.1192/bjp.bp.106.029009 18378982

[108] Rajji TK Ismail Z Mulsant BH. Age at onset and cognition in schizophrenia: meta-analysis. Br J Psychiatry. (2009) 195:286–93. doi: 10.1192/bjp.bp.108.060723 19794194

[109] Dittmann S Hennig-Fast K Gerber S Seemüller F Riedel M Severus WE. Cognitive functioning in euthymic bipolar I and bipolar II patients. Bipolar Disord. (2008) 10:877–87. doi: 10.1111/j.1399-5618.2008.00640.x 19594503

[110] Torres IJ Boudreau VG Yatham LN. Neuropsychological functioning in euthymic bipolar disorder: a meta-analysis. Acta Psychiatr Scand Suppl. (2007) 434):17–26. doi: 10.1111/j.1600-0447.2007.01055.x 17688459

[111] Kurtz MM Gerraty RT. A meta-analytic investigation of neurocognitive deficits in bipolar illness: profile and effects of clinical state. Neuropsychology. (2009) 23:551–62. doi: 10.1037/a0016277 PMC2804472 19702409

[112] Kapczinski F Magalhães PV Balanzá-Martinez V Dias VV Frangou S Gama CS. Staging systems in bipolar disorder: an International Society for Bipolar Disorders Task Force Report. Acta Psychiatr Scand. (2014) 130:354–63. doi: 10.1111/acps.12305 24961757

[113] van der Markt A Klumpers UM Draisma S Dols A Nolen WA Post RM. Testing a clinical staging model for bipolar disorder using longitudinal life chart data. BipolarDisord. (2019) 21:228–34. doi: 10.1111/bdi.12727 PMC6590317 30447123

[114] Strejilevich SA Samamé C Quiroz D. The neuroprogression hypothesis in bipolar disorders: Time for apologies? Bipolar Disord. (2023) 25:353–4. doi: 10.1111/bdi.13358 37578831

[115] Maziade M Rouleau N Mérette C Cellard C Battaglia M Marino C. Verbal and visual memory impairments among young offspring and healthy adult relatives of patients with schizophrenia and bipolar disorder: selective generational patterns indicate different developmental trajectories. Schizophr Bull. (2011) 37:1218–28. doi: 10.1093/schbul/sbq026 PMC3196959 20410238

[116] Lin A Yung AR Nelson B Brewer WJ Riley R Simmons M. Neurocognitive predictors of transition to psychosis: medium- to long-term findings from a sample at ultra-high risk for psychosis. Psychol Med. (2013) 43:2349–60. doi: 10.1017/s0033291713000123 23388122

[117] Bora E Yücel M Pantelis C. Neurocognitive markers of psychosis in bipolar disorder: a meta-analytic study. J Affect Disord. (2010) 127:1–9. doi: 10.1016/j.jad.2010.02.117 20231037

[118] Bora E Yücel M Pantelis C Berk M. Meta-analytic review of neurocognition in bipolar II disorder. Acta Psychiatr Scand. (2011) 123:165–74. doi: 10.1111/acps.2011.123.issue-3 21092023

[119] Lewandowski KE Cohen BM Ongur D. Evolution of neuropsychological dysfunction during the course of schizophrenia and bipolar disorder. Psychol Med. (2011) 41:225–41. doi: 10.1017/s0033291710001042 20836900

[120] Joseph MF Frazier TW Youngstrom EA Soares JC. A quantitative and qualitative review of neurocognitive performance in pediatric bipolar disorder. J Child Adolesc Psychopharmacol. (2008) 18:595–605. doi: 10.1089/cap.2008.064 19108664 PMC2768898

[121] Robinson LJ Thompson JM Gallagher P Goswami U Young AH Ferrier IN. Moore PB. A meta-analysis of cognitive deficits in euthymic patients with bipolar disorder. J Affect Disord. (2006) 93:105–15. doi: 10.1016/j.jad.2006.02.016 16677713

[122] Post RM Fleming J Kapczinski F. Neurobiological correlates of illness progression in the recurrent affective disorders. J Psychiatr Res. (2012) 46:561–73. doi: 10.1016/j.jpsychires.2012.02.004 22444599

[123] Samamé C Martino DJ Strejilevich SA. Longitudinal course of cognitive deficits in bipolar disorder: a meta-analytic study. J Affect Disord. (2014) 164:130–8. doi: 10.1016/j.jad.2014.04.028 24856566

[124] Schretlen DJ Peña J Aretouli E Orue I Cascella NG Pearlson GD. Confirmatory factor analysis reveals a latent cognitive structure common to bipolar disorder, schizophrenia, and normal controls. Bipolar Disord. (2013) 15:422–33. doi: 10.1111/bdi.12075 PMC4128168 23656284

[125] Krabbendam L Arts B van Os J Aleman A. Cognitive functioning in patients with schizophrenia and bipolar disorder: a quantitative review. Schizophr Res. (2005) 80:137–49. doi: 10.1016/j.schres.2005.08.004 16183257

[126] Bora E Yucel M Pantelis C. Theory of mind impairment in schizophrenia: meta-analysis. Schizophr Res. (2009) 109:1–9. doi: 10.1016/j.schres.2008.12.020 19195844

[127] Zanelli J Reichenberg A Sandin S Morgan C Dazzan P Pilecka I. Dynamic and static cognitive deficits in schizophrenia and bipolar disorder after the first episode. Schizophr Bull. (2022) 48:590–8. doi: 10.1093/schbul/sbab150 PMC9077411 35064259

[128] Czepielewski LS Massuda R Goi P Sulzbach-Vianna M Reckziegel R Costanzi M. Verbal episodic memory along the course of schizophrenia and bipolar disorder: a new perspective. Eur Neuropsychopharmacol. (2015) 25:169–75. doi: 10.1016/j.euroneuro.2014.09.006 25311898

[129] Rosa AR Magalhaes PV Czepielewski L Sulzbach MV Goi PD Vieta E. Clinical staging in bipolar disorder: focus on cognition and functioning. JClinPsychiatry. (2014) 75:e450–e6. doi: 10.4088/JCP.13m08625 24922497

[130] Gama CS Kunz M Magalhães PV Kapczinski F. Staging and neuroprogression in bipolar disorder: a systematic review of the literature. Braz J Psychiatry. (2013) 35:70–4. doi: 10.1016/j.rbp.2012.09.001 23567604

[131] Grande I Magalhães PV Chendo I Stertz L Panizutti B Colpo GD. Staging bipolar disorder: clinical, biochemical, and functional correlates. Acta Psychiatr Scand. (2014) 129:437–44. doi: 10.1111/acps.12268 24628576

[132] Peralta V Cuesta MJ. Schneider’s first-rank symptoms have neither diagnostic value for schizophrenia nor higher clinical validity than other delusions and hallucinations in psychotic disorders. Psychol Med. (2023) 53:2708–11. doi: 10.1017/s0033291720003293 32943125

[133] Yalınçetin B Ulaş H Var L Binbay T Akdede BB Alptekin K. Relation of formal thought disorder to symptomatic remission and social functioning in schizophrenia. Compr Psychiatry. (2016) 70:98–104. doi: 10.1016/j.comppsych.2016.07.001 27624428

[134] Andersen SM Randers A Jensen CM Bisgaard C Steinhausen HC. Preceding diagnoses to young adult bipolar disorder and schizophrenia in a nationwide study. BMC Psychiatry. (2013) 13:343. doi: 10.1186/1471-244x-13-343 24359146 PMC3898215

[135] Fountoulakis KN Popovic D Mosheva M Siamouli M Moutou K Gonda X. Mood symptoms in stabilized patients with schizophrenia: A bipolar type with predominant psychotic features? Psychiatr Danub. (2017) 29:148–54. doi: 10.24869/psyd.2017.148 28636572

[136] Aleman A Kahn RS. Strange feelings: do amygdala abnormalities dysregulate the emotional brain in schizophrenia? Prog Neurobiol. (2005) 77:283–98. doi: 10.1016/j.pneurobio.2005.11.005 16352388

[137] Planansky K Johnston R. Depressive syndrome in schizophrenia. Acta Psychiatr Scand. (1978) 57:207–18. doi: 10.1111/j.1600-0447.1978.tb06887.x 645411

[138] Borgmann-Winter K Willard SL Sinclair D Mirza N Turetsky B Berretta S. Translational potential of olfactory mucosa for the study of neuropsychiatric illness. Transl Psychiatry. (2015) 5:e527. doi: 10.1038/tp.2014.141 25781226 PMC4354342

[139] Bellon A Feuillet V Cortez-Resendiz A Mouaffak F Kong L Hong LE. Dopamine-induced pruning in monocyte-derived-neuronal-like cells (MDNCs) from patients with schizophrenia. Mol Psychiatry. (2022) 27:2787–802. doi: 10.1038/s41380-022-01514-w PMC9156413 35365810

[140] Liu YN Lu SY Yao J. Application of induced pluripotent stem cells to understand neurobiological basis of bipolar disorder and schizophrenia. Psychiatry Clin Neurosci. (2017) 71:579–99. doi: 10.1111/pcn.12528 28393474

[141] Watmuff B Berkovitch SS Huang JH Iaconelli J Toffel S Karmacharya R. Disease signatures for schizophrenia and bipolar disorder using patient-derived induced pluripotent stem cells. Mol Cell Neurosci. (2016) 73:96–103. doi: 10.1016/j.mcn.2016.01.003 26777134 PMC4867281

[142] Wei Y Wang T Li G Feng J Deng L Xu H. Investigation of systemic immune-inflammation index, neutrophil/high-density lipoprotein ratio, lymphocyte/high-density lipoprotein ratio, and monocyte/high-density lipoprotein ratio as indicators of inflammation in patients with schizophrenia and bipolar disorder. Front Psychiatry. (2022) 13:941728. doi: 10.3389/fpsyt.2022.941728 35958647 PMC9360542

